# Elucidating the Toxic Mechanism of NiWO_4_ to a Neotropical Microcrustacean: Experimental and Theoretical Insights

**DOI:** 10.1021/acsomega.5c08907

**Published:** 2025-11-27

**Authors:** Cínthia B de Abreu, Renan Castelhano Gebara, Thiago Trevizam Dorini, Giseli Swerts Rocha, Adrislaine da Silva Mansano, Marcelo Assis, Thalles Maranesi Pereira, Luciano Sindra Virtuoso, Miguel Angel San-Miguel, Maria da Graça Gama Melão, Elson Longo

**Affiliations:** † Center for the Development of Functional Materials (CDMF), 600424 Universidade Federal de São Carlos (UFSCar), Rodovia Washington Luís, Km 235, 13565-905 São Carlos, SP, Brazil; ‡ Instituto de Química, 28132 Universidade Estadual de Campinas, 13083-862 Campinas, São Paulo, Brasil; § Escola Tècnica Superior d’Enginyeria Química, Departament d’Enginyeria Química, Universitat Rovira i Virgili (URV), streetAv. Països Catalans, 26. 43007 Tarragona,Spain; ∥ Department of Hydrobiology, 600424 Universidade Federal de São Carlos (UFSCar), Rodovia Washington Luís, Km 235, 13565-905 São Carlos, SP, Brazil; ⊥ Department of Biosciences, 28105 Universidade Federal de São Paulo (UNIFESP), Rua Silva Jardim, 11015-020 Santos, Brazil; # Department of Chemistry, 74347 Universidade Federal de Alfenas (UNIFAL), Rua Gabriel Monteiro da Silva 700, 37130-001 Alfenas, MG, Brazil

## Abstract

In the present study, we describe the toxicity mechanism of nickel tungstate nanoparticles (NiWO_4_ NPs) and present experimental and computational advancements achieved through a combined approach to evaluate the toxicity of this semiconductor in the freshwater aquatic organism *Ceriodaphnia silvestrii* (Cladocera). Results from the acute toxicity test indicated that NiWO_4_ induced immobility in *C. silvestrii* at concentrations exceeding 40 mg L^–1^ (Dunn’s test, *p* < 0.05). The 48-h EC_50_ was 33.76 mg L^–1^ ± 5.67. The sublethal concentrations of NiWO_4_, at 20 and 25 mg L^–1^, significantly decreased the ingestion rates of *C. silvestrii* (Dunnett’s test, *p* < 0.05), directly affecting cladoceran nutrition and potentially hindering population growth. Density functional theory (DFT) and *ab initio* atomistic thermodynamics analyses characterized the structural, electronic, and energetic properties of low-index NiWO_4_ surfaces, revealing that oxygen-rich (111) facets may play a central role in oxidative stress and cellular damage in exposed organisms. Based on these findings, we propose a detailed toxicological mechanism that elucidates how nanoparticle properties influence biological responses in Neotropical microcrustaceans.

## Graphical abstract

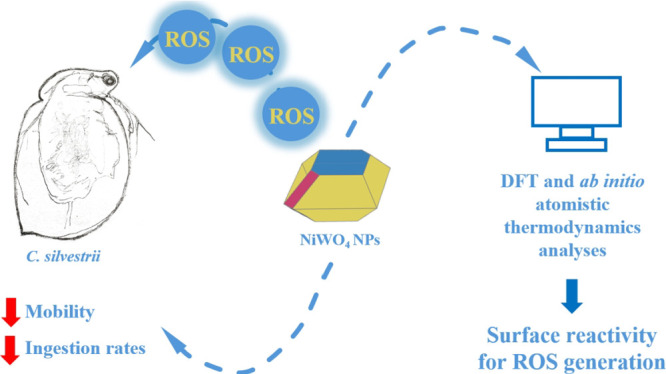

## Introduction

Nanoparticles (NPs) are increasingly detected across aquatic ecosystems, which often act as terminal sinks for these materials, raising concerns about exposure and biodiversity loss. Tungstate-based NPs merit particular attention because their compositions and surface chemistries can engender nontrivial ecotoxicological profiles.

Transition metal tungstates (MWO_4_) form robust frameworks in which [WO_*x*_] polyhedrons constitute the rigid lattice-forming units, while the transition metal (M) acts as a lattice modifier that tunes local bonding, defect chemistry, and electronic structure.^1^ Within this family, nickel tungstate (NiWO_4_) has attracted broad technological interest—including photocatalysis, photo/electrochemical energy conversion, pigments, gas/humidity sensing, and antimicrobial applications—because its defect chemistry and surface terminations can be engineered to modulate charge generation and interfacial reactions.^2–5^ These same properties are environmentally relevant: under illumination, NiWO_4_ generates reactive oxygen species (ROS) via photoexcitation and defect-mediated pathways, producing electron–hole pairs that drive ^·^OH and ^·^O_2_^–^ formation at water/oxygen-exposed surfaces.^2–6^

It is well established that semiconductor properties are strongly facet-dependent, because distinct exposed surfaces differ in atomic coordination, defect distributions, band alignment, and surface dipoles. Consequently, surface-specific structural and electronic analyses are essential for interpreting material function.^7–9^ Computationally, NP surfaces can be modeled with periodic slab models, quantifying stability via surface energies and, under realistic environments, via ab initio atomistic thermodynamics to obtain surface free energies as functions of chemical potentials (e.g., O_2_, H_2_O) and temperature. Wulff constructions relate facet-resolved surface free energies to equilibrium morphologies, while explicit or implicit solvation models approximate aqueous conditions relevant to ecotoxicology.^10–14^ Ecologically, semiconducting oxides such as NiWO_4_ can adversely affect microcrustaceans that occupy key positions in freshwater food webs, with impacts that may cascade to higher trophic levels and compromise ecosystem stability.^15–17^

Previous work from our group on the green microalga *Raphidocelis subcapitata* showed differential toxicity among tungstate NPs, with increasing toxicity in the order NiWO_4_ < CoWO_4_ < ZnWO_4_ < CuWO_4_.^18–21^ Despite the expanding technological use of NiWO_4_, data for Neotropical cladocerans and mechanistic links between NiWO_4_ surface chemistry, ROS generation, and organism-level end points remain unknown.

Here, we integrate ecotoxicological assays on *Ceriodaphnia silvestrii* with first-principles surface modeling and *ab initio* atomistic thermodynamics of NiWO_4_. We analyze how low-index facets and terminations modulate electronic structure and work function, and we correlate these predictions with acute and sublethal end points (immobility, feeding inhibition) in *C. silvestrii*. By coupling biological and computational evidence, we delineate surface-chemistry-dependent mechanisms for NiWO_4_ NP toxicity in freshwater microcrustaceans and refine broader principles of nanoparticle–biota interactions.

## Materials and Methods

### Synthesis and Characterization

NiWO_4_ NPs were synthesized using a coprecipitation method, followed by microwave-assisted hydrothermal treatment to enhance crystallinity and particle uniformity. Detailed procedures for the synthesis and characterization of the NPs are available in Abreu et al.^22^ The generation and identification of reactive oxygen species (ROS) induced by NiWO_4_ NPs are described in the Supporting Information.

### Microcrustacean Culture and Toxicity Tests

The cladoceran *C. silvestrii* was sourced from laboratory cultures at DEBE, Department of Ecology and Evolutionary Biology, Federal University of São Carlos (UFSCar, Brazil). The stock cultures were maintained at the Laboratory of Plankton, Department of Hydrobiology, Federal University of São Carlos (UFSCar, Brazil), in artificial water with a pH range of 7.0–7.6, conductivity of 160 μS cm^–1^, and hardness between 40 and 48 mg CaCO_3_ L^–1^. The organisms were cultured at a constant temperature of 25 ± 1 °C and under a 12:12 h light/dark photoperiod, following the guidelines established by the Brazilian Association of Technical Standards (ABNT NBR 13373, 2017). The organisms were fed thrice weekly with the microalga *Raphidocelis subcapitata* at a concentration of 2 × 10^5^ cells mL^–1^, supplemented with a mixture of yeast and fish food.^23^

For the toxicity tests, the stock solution (1000 mg L^–1^) was dispersed in ultrapure water using a bath sonicator (Ultra Cleaner 1400 Unique) for 30 min. The toxicity tests were conducted according to the guidelines established by the ABNT.^23,24^ For the toxicity test, the following nominal concentrations were tested: 0.0, 7.5, 10, 20, 30, 40, 50, 60, 70, 80, 90, and 100 mg L^–1^ of NiWO_4_. The acute toxicity assay was performed with four replicates per treatment, each containing five neonates (<24 h old), resulting in a total of 20 organisms per concentration. The tests were conducted in the dark at 25 ± 1 °C, without food supplementation. After 48 h of exposure, the number of immobile individuals was recorded, and the data were used to calculate the median effective concentration (48-h EC_50_).

Based on the results of the acute toxicity tests, feeding inhibition assays were conducted following the method described by^25^ at the following sublethal nominal concentrations: 0, 5, 10, 15, 20, and 25 mg L^–1^. Individuals of *C. silvestrii* (48 h old) were exposed to the sublethal concentrations of NiWO_4_ for 24 h in the dark and were fed with *R. subcapitata* at a concentration of 2 × 10^5^ cells mL^–1^. Each treatment had four replicates, with five animals per replicate (*n* = 20). An additional replicate, containing algae but no animals, was included to measure potential algal growth during the experiment. At the end of the 24-h exposure, the samples containing algal cells were fixed with 1% formaldehyde and analyzed using a flow cytometer (FACSCalibur, Becton Dickinson, San Jose, CA, USA), equipped with a 15 mW blue-argon ion laser (488 nm excitation). An internal standard of 6 μm fluorescent beads (Fluoresbrite carboxylate microspheres; Polysciences, Warrington, PA, USA) was used.^26^ The data were analyzed using FlowJo software, version 10 (Treestar, Woodburn, OR, USA). Ingestion rates were calculated according to the method of,^27^ with the relevant equations provided below ([Disp-formula eq1]–[Disp-formula eq3]).

$$F=\frac{V}{n}\times \frac{\ln\;C_{0}-\ln\;C_{t}}{t}-A$$ 1

$$A=\frac{\ln\;C_{0}-\ln\;C_{t}^{\prime }}{t}$$ 2

$$I=F\times \sqrt{C_{0}\times C_{t}}$$ 3

where: *F* is the filtration rate (mL ind^–1^ h^–1^), *I* is the ingestion rate (cells ind^–1^ h^–1^), *C*_0_ is the initial algae density (cells mL^–1^) at 0 h, *C*_*t*_ is the algae density (cells mL^–1^) at 24 h, *n* is the number of organisms per replicate, *V* is the volume of the test solution (mL), and *A* is the correction factor for changes in algal concentrations at 24 h (*C*_r_) in the treatments without animals.

### Computational Details

We performed all density functional theory (DFT) calculations using the Vienna Ab initio Simulation Package (VASP).^28–32^ The interactions between ions and electrons were described using the projector augmented-wave (PAW) method,^33^ and exchange-correlation effects were treated with the Perdew–Burke–Ernzerhof (PBE) generalized gradient approximation (GGA) functional.^34^ To accurately represent both core and valence electrons, we employed a plane-wave basis set with an energy cutoff of 500 eV. We note that the PBE-GGA functional has well-known limitations, including overestimation of lattice constants and underestimation of band gap energies.^35,36^ Nevertheless, trends in surface energies calculated with PBE-GGA are widely validated in the literature^37–40^ and provide reliable frameworks for comparative analysis of different surface configurations.

For the optimization of bulk and surface structures, we utilized a Γ-centered *k*-point mesh with a reciprocal space resolution of 0.2 Å^–1^. Atomic positions were relaxed until the forces on each atom were less than 0.01 eV/Å. To ensure convergence of surface properties, each surface slab was constructed with a minimum thickness of 15 Å. The top and bottom layers, each 7 Å thick, were allowed to relax, while the central region of the slab was kept fixed to simulate bulk-like behavior. The electronic self-consistency loop was converged until the total energy difference between successive iterations was less than 10^–5^ eV. All calculations and subsequent analysis were conducted using the PS- TEROS code,^41^ developed in our research group. This framework automates the entire workflow, from slab generation and VASP simulations to the thermodynamic analysis and plotting of surface stability, ensuring both consistency and reproducibility throughout the study.

### Data Analysis

The EC_50_ values were calculated by nonlinear regression with a sigmoidal 3-parameter logistic curve:^42^

$$Y=\frac{\max}{1+{\left(\frac{C}{{\mathrm{EC}}_{50}}\right)}^{\beta }}$$ 4

where *Y* is the response of the measured end point, max is the maximum observed response, *C* is the concentration of the tested substance, and β is the slope. The drc package in R,^43^ implemented in R version 4.0.5 using RStudio version 1.4.1717 ^44^ was used to calculate EC_10_ and EC_20_ values.^45^ Data from toxicity tests were first assessed for normality and homogeneity of variances. Normal data were then analyzed using one-way ANOVA, followed by Dunnett’s posthoc test. For data that did not follow a normal distribution, the Kruskal–Wallis test was applied, followed by Dunn’s posthoc test.

## Results and Discussion

### Characterization of NiWO_4_ NPs

The comprehensive characterization of NiWO_4_, including X-ray diffraction (XRD), Raman spectroscopy, and field emission scanning electron microscopy (FE-SEM) images, is detailed in our recent toxicity study of NiWO_4_ on the microalgae *R. subcapitata*,^20^ which was the first study regarding its toxicity to algae. Field emission scanning electron microscopy (FE-SEM) images (Figure S1 of the Supporting Information) revealed NiWO_4_ NPs with an irregular polyhedra morphology and an average particle size of 19.7 ± 4.6 nm.^20^ X-ray diffraction (XRD) analysis confirmed that the synthesized NiWO_4_ NPs possess a monoclinic crystal structure, corresponding to the *P*2/*c* space group, consistent with ICSD card no. 1879016.^46–48^ The absence of secondary phase peaks indicates the high phase purity of the material. The sharp and well-defined diffraction peaks further suggest a high degree of crystallinity. Complementary Raman spectroscopy analysis corroborated the XRD findings, revealing vibrational modes characteristic of the monoclinic wolframite-type NiWO_4_ structure.^6^

Table 1 summarizes the zeta potential, hydrodynamic size, and polydispersity index (PdI) data in both ultrapure water and artificial water. In artificial water, the zeta potential values ranged from −26.9 ± 0.7 to −31.03 ± 2.06 mV, suggesting that the suspension remains stable, as values near −30 mV are indicative of stability.^49^ Additionally, the polydispersity index (PdI) varied between 0.28 ± 0.06 and 0.63 ± 0.15, demonstrating that the particles exhibit a relatively narrow size distribution; with the lower values, an increase was observed with the increased NiWO_4_ concentration, indicating particle aggregation and resulting in larger sizes than those observed via microscopy. These findings contrast with those obtained when NiWO_4_ NPs were dispersed in the Oligo medium, a culture medium used to cultivate the microalga *R. subcapitata*.^18^ This discrepancy can be attributed to the influence of medium-specific factors on NP aggregation, including pH, concentration, ionic strength, and medium composition, as highlighted by Nogueira et al.^50^ These results highlight the importance of characterizing nanomaterials at different concentrations and under various exposure conditions to fully understand their behavior and toxicity.

**Table 1:** Characterization of NiWO_4_ NP in Ultrapure Water and Artificial Water at Different Concentrations of the Toxicity Test with *C. silvestrii*

	ultrapure water			artificial water		
NiWO_4_ (mg L^–1^)	hydrodynamic size (nm)	PdI	zeta potential (mV)	hydrodynamic size (nm)	PdI	zeta potential (mV)
5	253.40 ± 7.9	0.28 ± 0.03	–27.6 ± 1.6	281.1 ± 22.7	0.28 ± 0.065	–26.90 ± 0.7
7.5	156.77 ± 4.81	0.31 ± 0.03	–37.97 ± 0.21	224.87 ± 16.25	0.35 ± 0.31	–31.03 ± 2.06
10	157.57 ± 2.04	0.29 ± 0.02	–37.17 ± 0.55	292.50 ± 16.04	0.43 ± 0.10	–30.93 ± 0.75
15	175.40 ± 8.22	0.32 ± 0.00	–57.10 ± 1.0	236.53 ± 2.31	0.36 ± 0.33	–30.30 ± 1.04
20	149.60 ± 2.39	0.27 ± 0.03	–49.13 ± 0.57	260.70 ± 28.58	0.35 ± 0.01	–30.40 ± 0.72
25	149.33 ± 2.16	0.27 ± 0.01	–41.0 ± 6.02	369.67 ± 7.41	0.44 ± 0.06	–30.20 ± 0.75
30	150.23 ± 1.37	0.28 ± 0.03	–49.53 ± 1.01	702.87 ± 80.83	0.48 ± 0.04	–28.87 ± 0.32
40	159.90 ± 5.50	0.30 ± 0.02	–48.90 ± 1.15	864.23 ± 22.99	0.44 ± 0.05	– 29.57 ± 0.46
50	146.27 ± 1.27	0.25 ± 0.02	–53.23 ± 0.64	1977.67 ± 108.74	0.46 ± 0.12	–29.87 ± 0.67
60	151.00 ± 1.97	0.28 ± 0.01	–53.87 ± 0.80	2144.33 ± 248.60	0.56 ± 0.02	–27.73 ± 1.43
70	167.20 ± 2.55	0.33 ± 0.01	–51.90 ± 0.95	2281.33 ± 36.68	0.63 ± 0.13	–29.90 ± 0.96
80	174.40 ± 2.26	0.37 ± 0.02	–51.60 ± 1.0	2545.00 ± 177.65	0.65 ± 0.15	–28.87 ± 0.42
90	151.60 ± 7.01	0.27 ± 0.05	–53.73 ± 0.71	2813.67 ± 153.32	0.64 ± 0.04	–27.97 ± 1.46
100	142.00 ± 5.98	0.22 ± 0.03	–52.37 ± 0.55	2755.00 ± 96.38	0.54 ± 0.04	–29.20 ± 1.51

### Biological Results

Our toxicity tests were validated following ABNT standards,^23,24^ with mortality in the control group being less than 10%. In the acute toxicity test, we observed that NiWO_4_ induced immobility in *C. silvestrii* (Figure 1) at concentrations starting from 40 mg L^–1^ (Dunn’s test, *p* < 0.05), when compared to the control group. Regarding the EC_50_, our results indicated a mean 48-h EC_50_ value of 33.76 ± 5.67 mg L^–1^. The mean EC_20_ occurred at 27.26 ± 5.07 and EC_10_ 24.01 ± 4.7 mg L^–1^. The calculation of EC values is a key step in determining the levels at which a compound exerts toxic effects on organisms. Among these, the EC_10_ value is particularly valuable, as it can be used as a standard for water quality assessment^51^ and, for example, for generating species sensitivity distribution (SSD) curves.^42,52^ These values are especially useful in SSD development because they allow for direct comparisons of species sensitivity, enabling the identification of the most vulnerable organisms and the determination of environmentally protective concentration thresholds.^53^ As noted, this enhances the accuracy of ecological risk predictions and reinforces the scientific basis for environmental management strategies.

**Figure 1:**
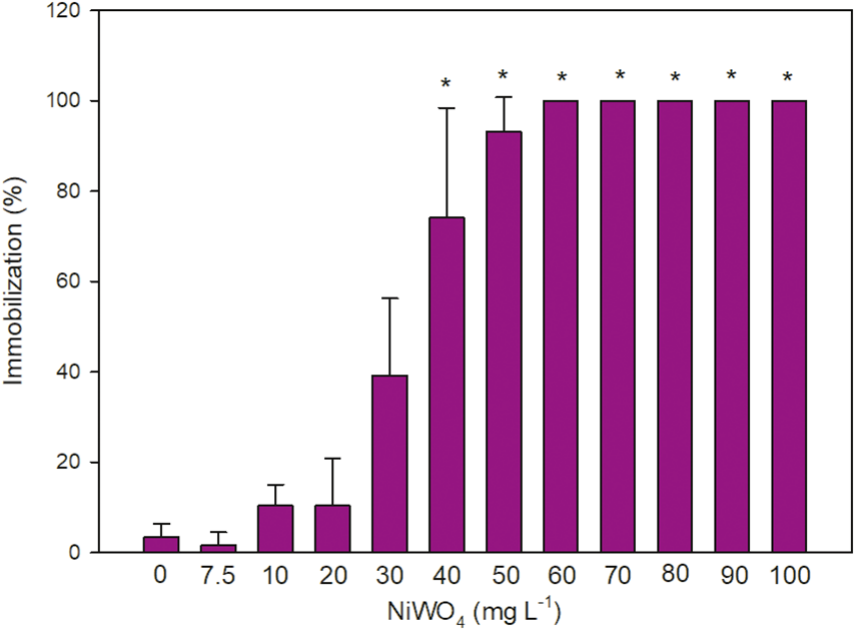
Immobility (%) of *C. silvestrii* (mean ± SD) after 48 h of exposure to NiWO_4_ NPs. Asterisks (*) represent significant differences from the control group (*p* < 0.05). The control group is represented by the number “0”.

To our knowledge, no studies have evaluated the toxicity of Ni NPs on *C. silvestrii*. There are a few studies on the toxicity of Ni NPs in aquatic organisms. Nogueira et al.^50^ reported a 48-h EC_50_ value of 14.6 mg L^–1^ for *Daphnia magna* exposed to NiO NPs. Conversely, another study by Nogueira et al.,^54^ which investigated the effects of Ni nanowires on *D. magna*, found no harmful effects in a lethal toxicity test.

Griffitt et al.^55^ investigated the toxicity of Ni NPs to *C. dubia* and reported a 48-h LC_50_ of 0.674 mg L^–1^. This value is approximately 50 times lower (i.e., more toxic) than the 48-h EC_50_ determined in the present study for *C. silvestrii*, underscoring potential species-specific sensitivity and the enhanced toxicity of Ni in NPs. This disparity highlights the critical need for further ecotoxicological studies on different species and NP formulations. The current literature also shows differences in the reported effects of Ni on microcrustaceans. For example, Pane et al.^56^ found that exposure to 0.085 mg L^–1^ of dissolved Ni significantly reduced survival, reproduction, and growth in *D. magna*. In contrast, Griffitt et al.^55^ reported a higher 48-h LC_50_ of 19.64 mg L^–1^ for *C. dubia* when exposed to soluble Ni, suggesting considerable variation in Ni sensitivity across species and experimental conditions. These discrepancies reinforce the importance of standardized testing protocols and comparative studies across taxa to better understand the ecological risks associated with Ni exposure.

The sublethal concentrations of NiWO_4_, at 20 and 25 mg L^–1^, significantly decreased (Dunnett’s test, *p* < 0.05) the ingestion rates of *C. silvestrii* (Figure 2). It is known that NPs can adhere to the gastrointestinal tract and compromise nutrient absorption,^15,16,50,54^ and have low clearance.^57,58^ Lu et al.^57^ point out that filtration and ingestion rates were consistent with the inhibition of reproduction and growth of *Daphnia*, and that this decrease in food intake due to NP exposure may be a cause of chronic toxicity. From this perspective, subsequent research will focus on assessing the chronic toxicity of NiWO_4_ NPs in Neotropical aquatic species, specifically investigating potential impacts on reproductive and fertility end points, in alignment with the findings of Lu et al.^57^

**Figure 2:**
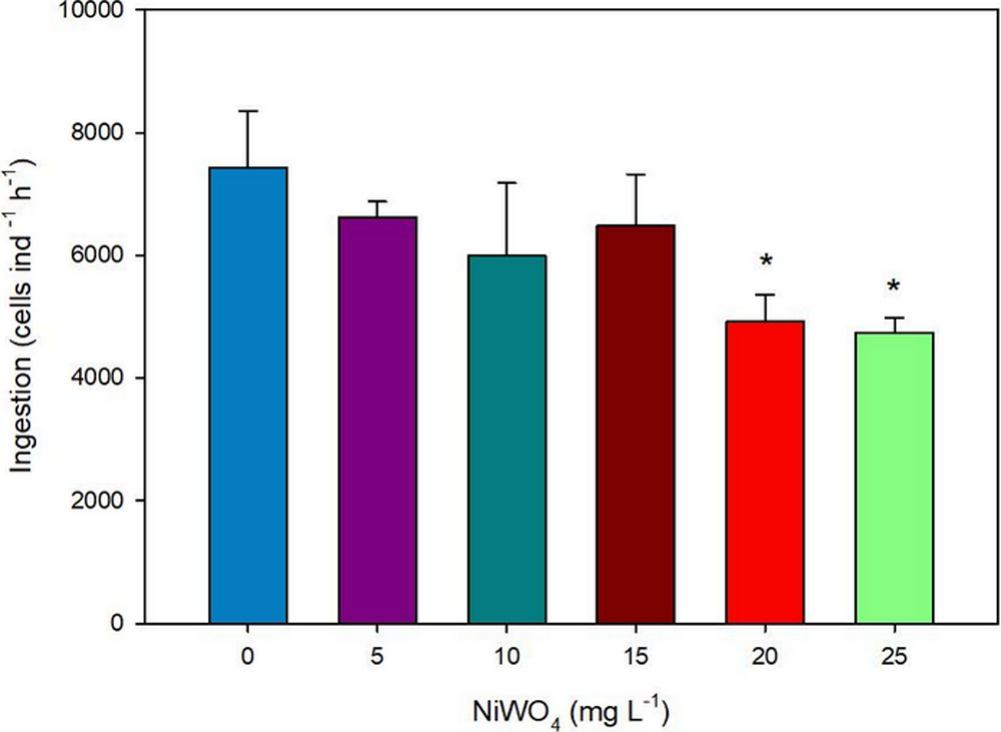
Ingestion rates (cells ind^–1^ h^–1^) of *C. silvestrii* (mean ± SD) exposed to NiWO_4_ NPs for 24 h. Asterisks (*) represent significant differences from the control group (*p* < 0.05). The control group is represented by the number “0”.

In the present study, the toxic effects of NiWO_4_ NPs on *C. silvestrii* can be attributed to the ROS released by the NPs, as demonstrated and discussed in section Toxicity Mechanism, which suggests that the production of ROS by NiWO_4_ is likely the primary factor contributing to its toxicity. In light of these observations, we have proposed and experimentally demonstrated a mechanism that provides more specific insights into the role of oxidative stress in nickel nanoparticle-induced toxicity.

NPs are known to interact with organic components in aquatic environments, which can alter the availability of these substances to organisms within the ecosystem.^58^ Exposure to NPs can cause various forms of cellular damage in organisms, including disruption of cell membranes and nucleic acids (DNA and RNA), overproduction of ROS, oxidative stress, and impairment of electron transport during cellular respiration.^59,60^ These effects can significantly compromise physiological and metabolic processes. In microcrustaceans of the order Cladocera, NP exposure has been linked to several adverse outcomes, such as immobility^61,62^ and mortality,^62^ as observed here; increased ROS production;^52,61^ and metabolic alterations, including changes in ingestion rates.^52^

Our research reveals a close relationship between the end points evaluated here, making them sensitive indicators for assessing nanoparticle toxicity. From an ecological perspective, the toxic effects of NiWO_4_ on the survival and ingestion rates of microcrustaceans could directly disrupt ecosystem balance. Furthermore, these organisms play a crucial role in linking primary producers, such as phytoplankton, to herbivores, and, at higher trophic levels, serve as prey for fish and larger vertebrates. As such, they are vital indicators of ecosystem changes. Moreover, our study advances the understanding of the toxicity of Ni-based NPs and enhances the Ni NP toxicity database, thereby supporting the development of appropriate regulatory recommendations for nickel NPs in surface waters, a gap highlighted by Meyer et al.^63^ These authors emphasize the importance of increasing the number of studies assessing the toxicity of Ni NPs.

### Toxicity Mechanism

The primary driver of NiWO_4_ NPs toxicity is the ROS generation. NiWO_4_ is a p-type semiconductor with a band gap of ∼2.8–3.9 eV, so it absorbs only the near-UV/blue portion of light (≤450 nm). Intrinsic defects and surface states can host charges that sustain low-level ROS formation even in the dark. Under visible-light irradiation, ROS production rises sharply because photoexcited electron–hole pairs react with water and dissolved oxygen to produce hydroxyl radicals (^·^OH) and superoxide (^·^O_2_^–^).^6^ This photoactivity explains the reported photocatalytic and antimicrobial behaviors of NiWO_4_, although its ROS output is typically lower than that of benchmark photocatalysts. To test for photoinduced ROS under exposure conditions relevant to microcrustacean culture, we performed scavenger assays while monitoring the photocatalytic discoloration of Rhodamine B (RhB) by NiWO_4_ NPs. Figure 3a shows that photocatalytic efficiency is reduced by the addition of all scavengers used, being more prominent for photogenerated holes (h^+^) originating from the excitation of e^–^ in basal states, as well ^·^as ^·^O_2_^–^ and ^·^OH radicals.

**Figure 3:**
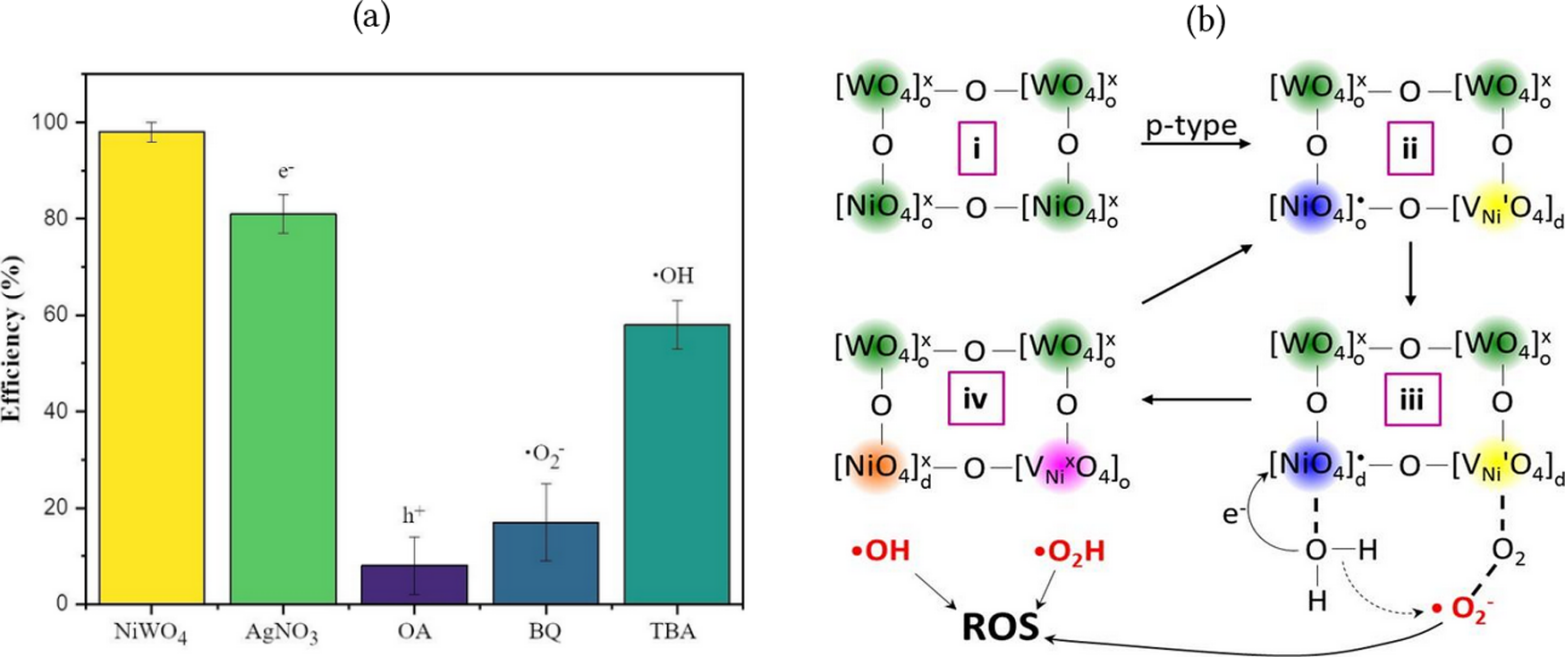
(a) Scavenger tests for photodegradation of RhB mediated by NiWO_4_ NPs; (b) mechanism of ROS production by NiWO_4_ NPs.

Figure 3b summarizes the proposed cycle of ROS generation by NiWO_4_ nanoparticles. (i) NiWO_4_ is composed of ordered clusters of [WO_6_] and [NiO_6_] connected by oxygen bridges. (ii) During synthesis, nickel vacancies are generated spontaneously, resulting in a p-type semiconductor with some local structural disorder. These defects increase the population of electrons in the conduction band and may slightly decrease the band gap. (iii) At the nanoparticle surface, these clusters readily interact with H_2_O and O_2_ at the same time. Under visible light, holes in the valence band oxidize H_2_O, producing ^·^OH and H^+^, and the corresponding electrons reduce adsorbed O_2_ to ^·^O_2_^–^. The latter can then react with H^+^ to form the hydroperoxyl radical (^·^OOH). (iv) After these redox steps, the system returns to electroneutrality and the cycle can begin again. Recently, we also demonstrated that the coadsorption of O_2_ and H_2_O molecules on the Ag_3_PO_4_(110) surface, through a synergistic mechanism, can produce ^·^OH and ^·^O^–^ species with lower energy barriers than those from the separate molecules, thereby enhancing ROS generation and, consequently, oxidative activity.^64^

## Thermodynamic Stability of NiWO_4_ Surfaces

In parallel with our experimental assessment of NiWO_4_ toxicity, we performed a theoretical investigation to elucidate the surface structure, thermodynamics, and reactivity of the clusters observed in the experiments. Using DFT-based calculations, we examined various surface terminations and their energetics to reveal how subtle differences in atomic arrangement can influence electron transfer and reactive oxygen species (ROS) generation. This analysis provides a mechanistic framework that links the material’s surface properties to its observed toxic effects in microcrustaceans.

### Structure Models

NiWO_4_ crystallizes in the monoclinic *P*2/*c* space group (Materials Project identifier: mp-21179 ^65^), adopting a zeta iron carbide-derived structure. In this arrangement, W^6+^ cations coordinate with six O^2–^ anions to form distorted WO_6_ octahedra. These octahedra share corners with eight equivalent NiO_6_ octahedra and edges with two equivalent WO_6_ octahedra, with corner-sharing octahedral tilt angles ranging from 48° to 56°. The W–O bond lengths vary between 1.81 and 2.12 Å. Similarly, Ni^2+^ cations form NiO_6_ octahedra by bonding with six O^2–^ anions. These NiO_6_ octahedra share corners with eight equivalent WO_6_ octahedra and edges with two equivalent NiO_6_ octahedra, exhibiting Ni–O bond lengths from 2.03 to 2.06 Å. There are two distinct O^2–^ sites: the first bonds in a distorted trigonal planar geometry to two W^6+^ cations and one Ni^2+^ cation, while the second bonds to one W^6+^ and two Ni^2+^ cations in a similar geometry.

Our optimized parameters for NiWO_4_ are *a* = 4.611 Å, *b* = 5.709 Å, *c* = 4.961 Å, and β = 89.86°, which closely match the values reported in the Materials Project database.^65^ Using this optimized bulk structure, we generated surface models by cleaving along the (100), (110), and (111) planes to represent the low-index surfaces of the crystal. The SlabGenerator class from the Pymatgen module was employed to obtain several possible symmetric slabs.

To thoroughly investigate the structural and electronic properties associated with the ordering and disordering of Ni^2+^ and W^6+^ cations at various surface terminations, Figure 4 presents schematic 3D and 2D representations of the NiWO_4_ crystal along the (a) (100), (b) (110), and (c) (111) surfaces. We identified 20 unique terminations—6 for the (100), 7 for the (110), and 7 for the (111) surfaces—featuring coordinated and undercoordinated [NiO_6_] and [WO_6_] clusters. The terminations are categorized based on the types of atoms and ions exposed at the surface into seven distinct types: (i) Ni, (ii) W, (iii) O, (iv) Ni/O, (v) Ni/W, (vi) W/O, and (vii) Ni/W/O. Specifically, the (100) surface exhibits O, Ni, and W terminations; the (110) surface includes O, Ni/W/O, Ni/W, and Ni/O terminations; and the (111) surface presents O, W/O, and Ni/W/O terminations. To distinguish between multiple terminations with similar exposed atoms, each termination is uniquely labeled according to its surface and sequence (e.g., A1, A2, A3 for the (100) surface).

**Figure 4:**
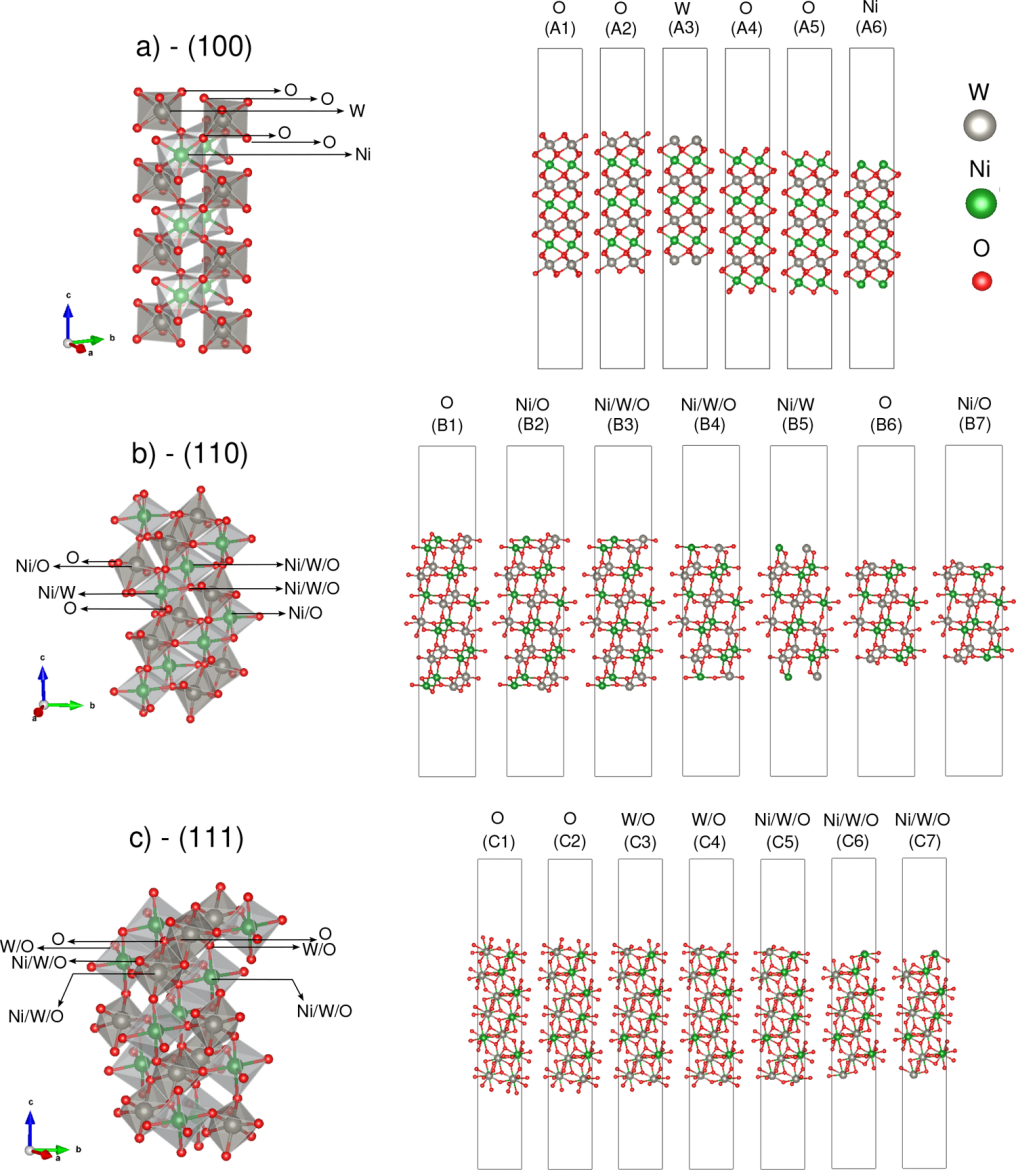
Representation of the structure models (A–C) for the (100), (110), and (111) orientations, respectively, of the NiWO_4_ unrelaxed surfaces. Ni, W, and O atoms are represented by green, silver, and red spheres, respectively.

### Surface Stability

To examine the stability of various terminations of the NiWO_4_ (110), (111), and (011) surfaces, we applied the ab initio atomistic thermodynamics approach as formulated by Rogal and Reuter.^37,66–68^ The effectiveness of this methodology has been thoroughly validated across diverse oxide systems, spanning from simple binary oxides such as RuO_2_(110)^37^ and In_2_O_3_(111),^69^ to more intricate ternary compounds including β-Ag_2_MoO_4_ (111), (011), and (100),^38^ BaTiO_3_ (001)^70^ ZnWO_4_(100),^39^ and ZnV_2_O_6_(001).^40^ A comprehensive description of this method is provided in the Supporting Information (SI).

We start by calculating the surface Gibbs free energy (γ) for various NiWO_4_ surface terminations as functions of the oxygen chemical potential (Δμ_O_) and temperature, while keeping the nickel chemical potential constant at Δμ_Ni_ = 0 eV. The calculated results are presented in Figure 5a. Our analysis identifies the most stable terminations in the following order: A5, C1, B2, C2, and C5. When the stoichiometry of the slab matches that of the bulk—thus preserving the W–O bonds—γ remains unaffected by changes in Δμ_O_. For example, as shown in Table 2, terminations C5 and B4 have (*N*_O_ – 4*N*_W_) = 0, confirming that the [WO_6_] clusters are intact, which results in γ being independent of Δμ_O_.

**Table 2:** Excess of Ni and O Atoms in Surface Terminations with Respect to W atoms, and the θ Value of Different Surface Terminations for the (100), (110), and (111) Surfaces of NiWO_4_

surface	termination	(*N*_O_ – 4*N*_W_)	(*N*_Ni_ – *N*_W_)	θ (eV/Å^2^)
(100)	A1	0	–2	7.38
	A2	–4	–2	15.31
	A3	–8	–2	34.59
	A4	8	2	7.31
	A5	4	2	2.55
	A6	0	2	7.08
(110)	B1	4	0	7.69
	B2	2	0	5.05
	B3	0	0	10.12
	B4	0	0	8.29
	B5	–2	0	12.46
	B6	–4	0	19.18
	B7	–4	0	18.45
(111)	C1	6	0	6.39
	C2	4	0	9.09
	C3	2	0	10.52
	C4	2	0	11.07
	C5	0	0	9.67
	C6	–4	0	18.82
	C7	–6	0	26.56

**Figure 5:**
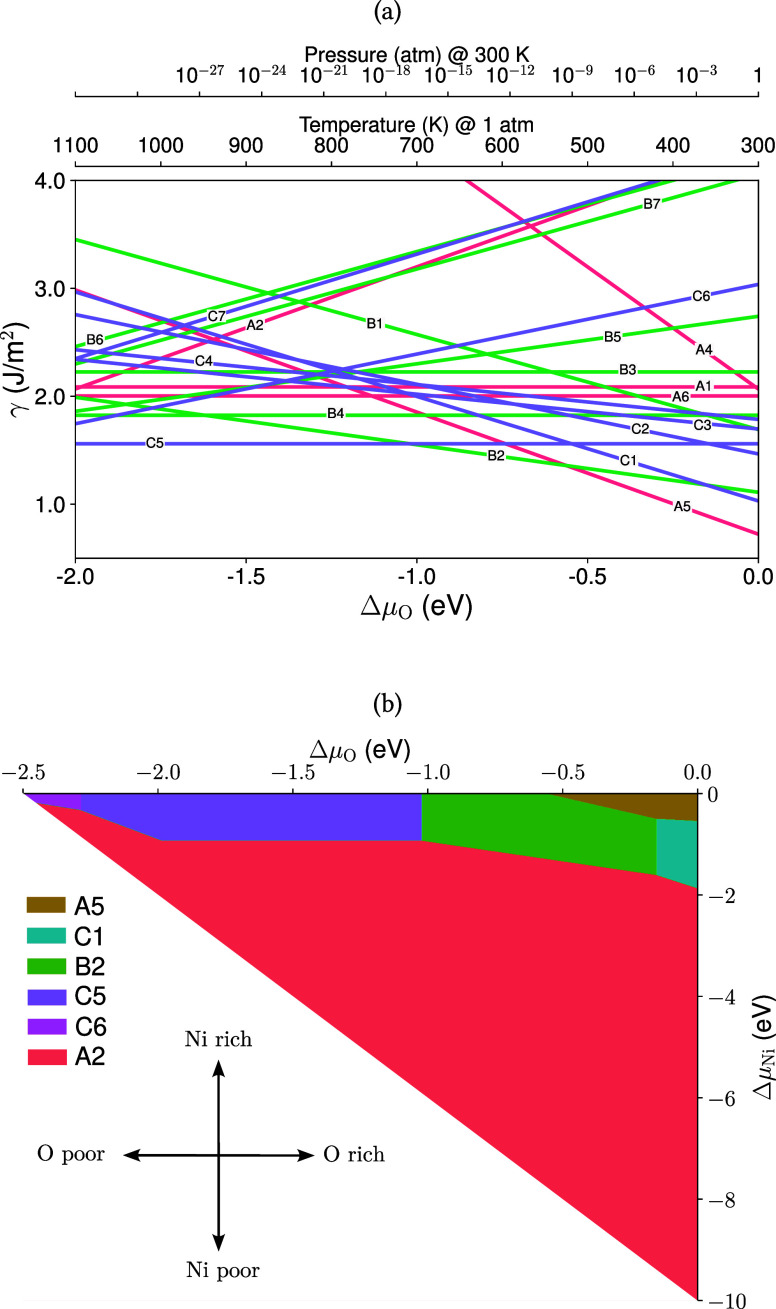
(a) Surface Gibbs free energy (γ) as a function of oxygen chemical potential (Δμ_O_) for various NiWO_4_ surface terminations at fixed Δμ_Ni_ = 0 eV. Each line represents a distinct surface termination labeled according to Figure 4. The top axis shows the corresponding oxygen partial pressure at *T* = 300 K, and the bottom axis shows temperature at *P* = 1 atm, both related to Δμ_O_ through the ideal gas approximation: Δμ_O_ = *kT*ln­(*p*/*p*_0_). (b) Phase diagram showing the stability regions of different NiWO_4_ surface terminations as a function of Δμ_Ni_ and Δμ_O_.

When (*N*_O_ – 4*N*_W_) deviates from zero, the slab’s stoichiometry differs from the bulk, leading to an oxygen deficiency or excess relative to tungsten. Negative values of (*N*_O_ – 4*N*_W_), as seen in terminations A2 (−4) and C6 (−4), indicate an oxygen deficiency. This causes a positive slope in the γ versus Δμ_O_ plot, meaning the slab becomes more stable as Δμ_O_ increases. Conversely, positive values of (*N*_O_ – 4*N*_W_), such as in terminations A5 (4), B2 (2), and C1 (6), indicate an oxygen excess, resulting in a negative slope and decreased slab stability with increasing Δμ_O_.

The θ parameters listed in Table 2 represent the energetic deviation of the slab from the bulk structure. Lower θ values—for instance, A5 (2.55 eV/Å^2^), B2 (5.05 eV/Å^2^), and C1 (6.39 eV/Å^2^)—indicate minimal deviation and thus higher stability. Specifically, the low θ value of A5 signifies strong alignment with the bulk structure, reinforcing its stability.

Phase boundaries between different terminations are established by solving the equation γ_*i*_ = γ_*j*_, which identifies the conditions under which one termination becomes more stable than another. These boundaries are illustrated in Figure 5b. Under low Δμ_Ni_ and low Δμ_O_ conditions, the stable competing phases are A5, C1, and B5. As Δμ_O_ increases, terminations with higher (*N*_O_ – 4*N*_W_) values, such as C5 and C6, become more favorable. At higher values of Δμ_Ni_, the A2 termination emerges as the most stable across the phase diagram, indicating a preference for the (100) surface under elevated nickel chemical potentials. Additionally, at increased Δμ_O_, terminations with negative (*N*_O_ – 4*N*_W_) become more stable due to the compensation of oxygen deficiency by the higher oxygen chemical potential.

## Surface Morphology and Reactivity

To explore the link between surface structure and reactivity in NiWO_4_, we used Wulff construction to determine its equilibrium crystal morphology and calculated work functions to assess its electronic properties. This combined approach reveals how variations in surface geometry and composition affect electron transfer and reactive oxygen species (ROS) formation, which may contribute to the toxicity observed in microcrustaceans.

Figure 6 presents the equilibrium crystal morphology of NiWO_4_ at two distinct oxygen chemical potential values (Δμ_O_ = 0 eV and Δμ_O_ = −2 eV), alongside the minimum surface energies for each crystallographic orientation. The Wulff construction reveals a predominance of the (111) facets (yellow) at both chemical potential values, with significant differences in the relative expression of (100) and (110) facets. At Δμ_O_ = 0 eV, the (100) facets (blue) constitute a substantial portion of the exposed crystal surface, whereas at Δμ_O_ = −2 eV, these facets are dramatically reduced in area, with a corresponding increase in the expression of (111) facets.

**Figure 6:**
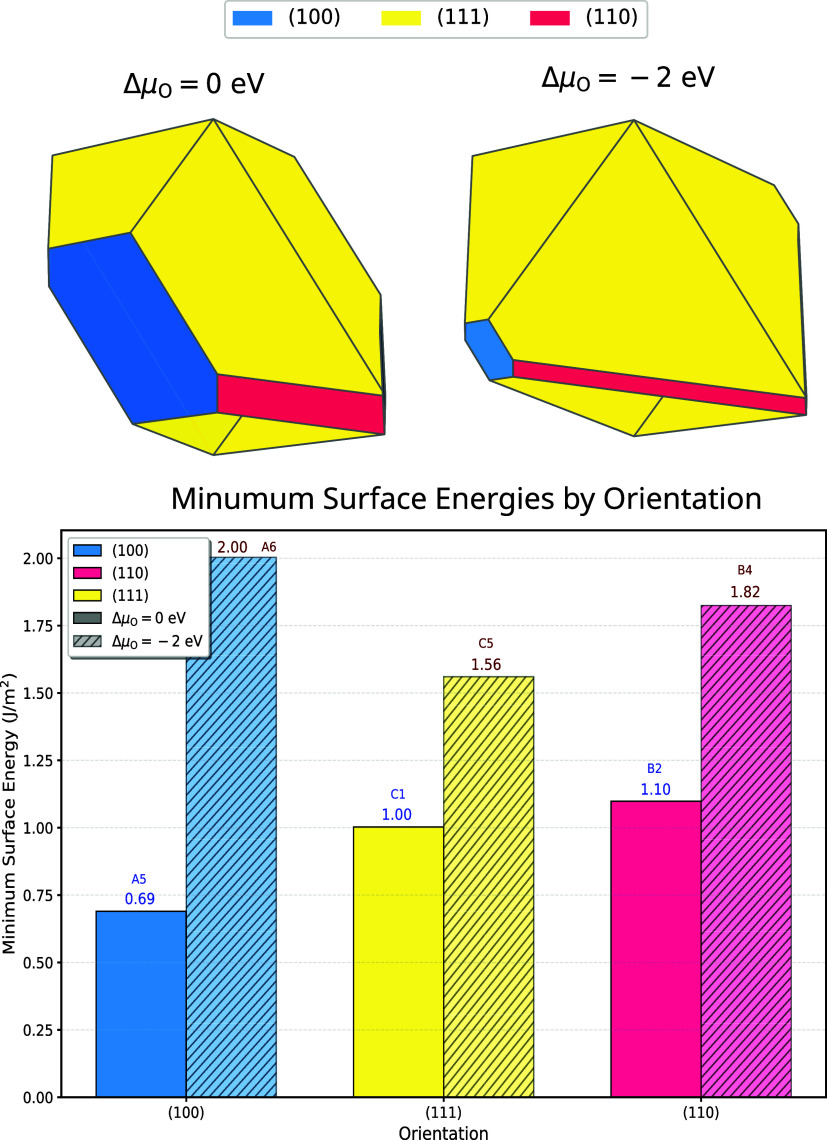
Equilibrium crystal morphology of NiWO_4_ determined via Wulff construction at two oxygen chemical potential values (Δμ_O_ = 0 eV and Δμ_O_ = −2 eV) (top). Minimum surface energies for the (100), (111), and (110) orientations under the same chemical potential conditions (bottom). The colors correspond to different crystallographic orientations: blue for (100), yellow for (111), and pink for (110).

The minimum surface energies exhibit a clear dependence on both crystallographic orientation and oxygen chemical potential. At Δμ_O_ = 0 eV, the (100) orientation displays the lowest surface energy (0.69 J/m^2^ for termination A5), followed by the (111) orientation (1.00 J/m^2^ for termination C1) and the (110) orientation (1.10 J/m^2^ for termination B2). However, at reduced oxygen chemical potential (Δμ_O_ = −2 eV), the surface energies increase substantially across all orientations, with the (100) orientation exhibiting the most dramatic change (2.00 J/m^2^ for termination A6), suggesting significant oxygen-dependent surface stability.

To investigate the electronic properties that govern surface reactivity, we calculated the work function for various surface terminations, categorized by the exposed atomic species. Figure 7 illustrates the average work function and corresponding surface free energy as a function of termination type. A clear trend emerges wherein oxygen-rich terminations (O, W/O, Ni/O) exhibit significantly higher work functions (6.9, 6.3, and 6.3 eV, respectively) compared to metal-rich terminations (W, Ni/W, Ni) which display progressively lower values (4.8, 4.8, and 4.5 eV, respectively). This trend is consistent with the electronegativity of the surface species, with oxygen-terminated surfaces inducing a more substantial surface dipole that increases the energy required for electron extraction.

**Figure 7:**
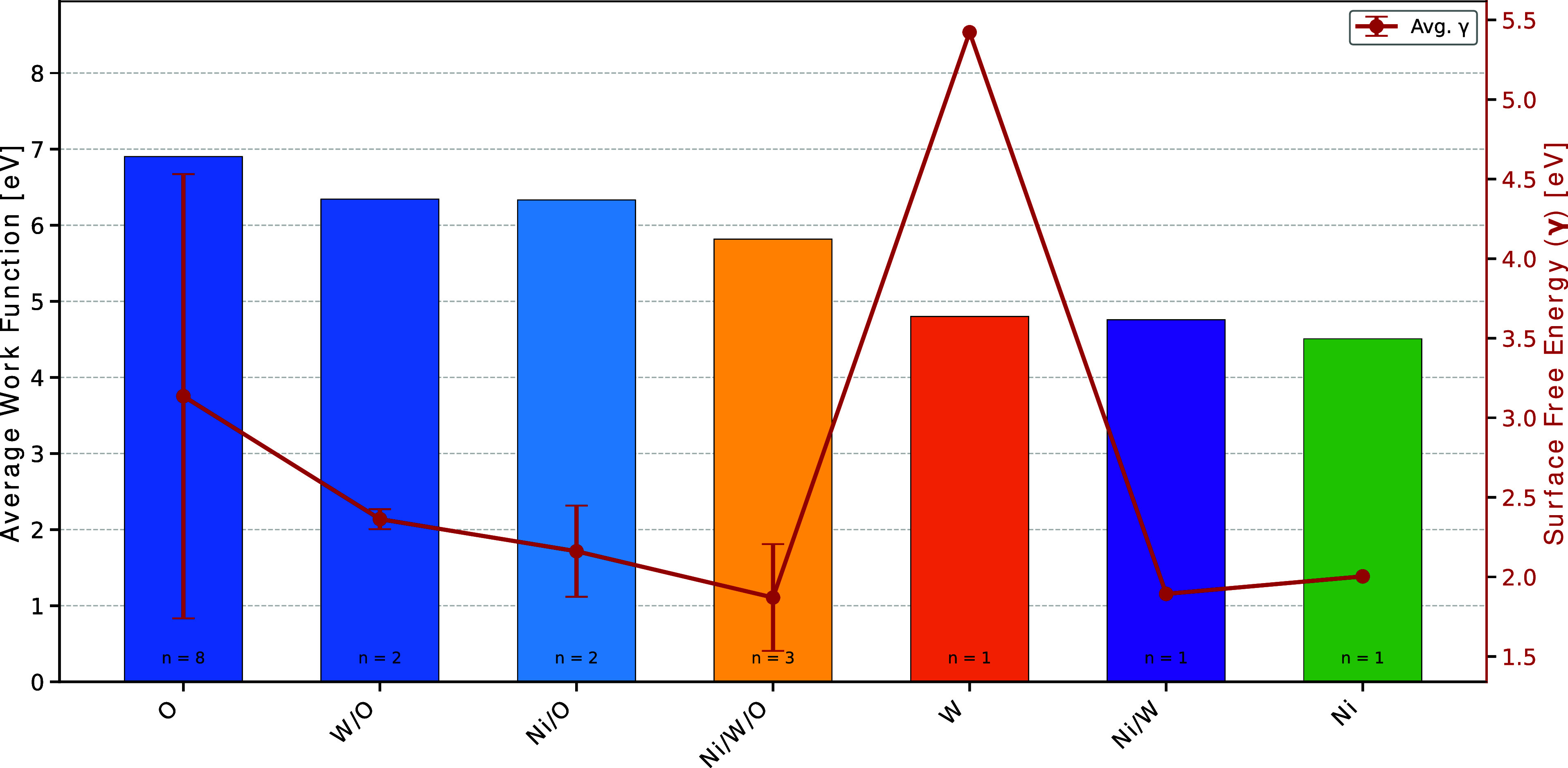
Average work function (bars, left axis) and surface free energy (red line, right axis) as a function of surface termination type in NiWO_4_. Error bars represent the standard deviation within each termination. The number of distinct terminations analyzed in each category is indicated at the bottom of each bar (*n*).

The tungsten-terminated surfaces (W) exhibit anomalous thermodynamic behavior characterized by substantially elevated surface energy values. This phenomenon can be attributed to the energetic unfavorability of cleaving the [WO_6_] octahedral clusters, which disrupts the structural integrity of the crystal lattice. This observation aligns with findings from similar ternary oxide systems such as β-Ag_2_MoO_4_,^38^ where the cleavage of [MoO_4_] tetrahedral clusters was demonstrated to be energetically unfavorable compared to the cleavage of metal–oxygen bonds associated with Ag^+^ cations.

Analysis of the surface thermodynamic stability in conjunction with work function measurements provides critical insights into the electronic properties of the most prevalent exposed facets. The predominant (111) facets with moderate work functions (5.9–7.0 eV for terminations B1, B7, and B8) facilitate appropriate band alignments for electron transfer processes involved in ROS generation. The higher work functions associated with oxygen-terminated surfaces enhance hole-mediated oxidation reactions with adsorbed water molecules, while the lower work functions of metal-rich terminations facilitate electron transfer to oxygen molecules.

The observed correlation between work function and surface termination provides critical mechanistic insights into the potential reactive oxygen species (ROS) generation processes detailed in section Toxicity Mechanism. Specifically, the lower work function of metal-rich terminations might facilitates electron transfer to adsorbed molecular oxygen, promoting the formation of superoxide radicals (^·^O_2_^–^) as depicted in Figure 3b. Conversely, the higher work function of oxygen-terminated surfaces enhances the ability to extract electrons from adsorbed water molecules, generating hydroxyl radicals (^·^OH) and protons (H^+^).

Furthermore, the significant morphological changes observed between different oxygen chemical potential conditions indicate that NiWO_4_ nanoparticles may exhibit environment-dependent toxicity mechanisms. In oxygen-rich environments, the increased expression of (111) facets with moderate work functions could enhance the generation of superoxide and hydroxyl radicals, thereby intensifying oxidative stress in exposed organisms. Our computational findings elucidate part of the complex mechanisms involved in the experimental observations of immobility and impaired fertility in *C. silvestrii*, where ROS-mediated oxidative damage was identified as the primary toxicity mechanism.

## Conclusions

This study provides the first comprehensive assessment of the toxicity mechanisms of NiWO_4_ NPs on the freshwater microcrustacean *C. silvestrii*, integrating both experimental and computational approaches. Our findings demonstrate that NiWO_4_ NPs induce acute and sublethal toxic effects, including immobility at 40 mg L^–1^ and reduced ingestion rates at 20 and 25 mg L^–1^. Additionally, the observed environment-dependent morphological variations in NiWO_4_, particularly under oxygen-rich conditions, suggest that the toxicity of these NPs is modulated by their surface chemistry and crystal facet expression. Computational modeling revealed that enhanced generation of ROS, driven by specific surface facets such as (111), plays a central role in oxidative stress and cellular damage in exposed organisms. Together, these results advance our understanding of NiWO_4_ NP toxicity and highlight the importance of combining biological and computational tools to unravel nanoparticle–organism interactions in aquatic systems.

## Supplementary Material



## Notes

Synthesis and characterization of NiWO_4_; field emission scanning electron microscopy; and surface Gibbs free energy analysis (PDF)
