# Supplementary material for: Elucidating the Toxic Mechanism of NiWO4 to a Neotropical Microcrustacean: Experimental and Theoretical Insights
Source: ACS Omega. 2025 Nov 27;10(48):59413–24. doi: 10.1021/acsomega.5c08907 (PMC12784302; doi:10.1021/acsomega.5c08907)
Supplement: Supplementary file 1 [file ao5c08907_si_001.pdf]

Supplementary Material: Elucidating the toxic mechanism of $NiWO_4$ to a Neotropical microcrustacean: experimental and theoretical insights

Cínthia B. de Abreu,[*,†] Renan Castelhano Gebara,[†] Thiago Trevizam Dorini,[‡] Giseli Swerts Rocha,[¶] Adrislaine da Silva Mansano,[§] Marcelo Assis,[‖] Thalles Maranesi Pereira,[⊥] Luciano Sindra Virtuoso,[⊥] Miguel Angel San-Miguel,[‡] Maria da Graça Gama Melão,[§] and Elson Longo[†]

†*Center for the Development of Functional Materials (CDMF), Universidade Federal de São Carlos (UFSCar), Rodovia Washington Luís, Km 235, 13565-905, São Carlos, SP, Brazil.*

‡*Instituto de Química, Universidade Estadual de Campinas, 13083-970, Campinas, São Paulo, Brazil.*

¶*Universitat Rovira i Virgili (URV), Escola Tècnica Superior d'Enginyeria Química, Departament d'Enginyeria Química, Av. Països Catalans, 26. 43007, Tarragona, Spain.*

§*Department of Hydrobiology, Universidade Federal de São Carlos (UFSCar), Rodovia Washington Luís, Km 235, 13565-905, São Carlos, SP, Brazil.*

‖*Department of Biosciences, Universidade Federal de São Paulo (UNIFESP), Rua Silva Jardim, Santos 11015-020, Brazil.*

⊥*Department of Chemistry, Universidade Federal de Alfenas (UNIFAL), Rua Gabriel Monteiro da Silva 700, Alfenas, MG, 37130-001, Brazil.*

E-mail: cinthia.abreu123@gmail.com

Supplementary Material

Synthesis and Characterization

$NiWO_4$ NPs were synthesized using the coprecipitation method followed by microwave-assisted hydrothermal irradiation. Initially, two solutions were prepared: (i) 3.3318 g of sodium tungstate ($Na_2WO_4 \cdot 2H_2O$, Sigma-Aldrich, 99.9% purity) in 50 mL of distilled water, and (ii) 2.9079 g of nickel(II) nitrate hexahydrate ($Ni(NO_3)_2 \cdot 6H_2O$, Sigma-Aldrich, 98.5% purity) in 50 mL of distilled water. Solution (ii) was added to solution (i) under constant stirring, resulting in a green precipitate. This system was stirred for 10 minutes to enhance system homogeneity and then transferred to a Teflon reactor, sealed, and placed in the microwave hydrothermal system (2.45 GHz, maximum power of 800 W) at a temperature of 160°C for 32 minutes. Following this process, the green precipitate was washed with water ten times, centrifuged, and then dried at 60°C for 12 h. Once this powder was ready, it underwent a thermal treatment in a conventional muffle furnace at 550°C for 2 hours, using a ramp of 10°C/min, resulting in a dark yellow powder.

The sample was characterized by X-ray diffraction (XRD) using a D/Max-2500PC diffractometer (Rigaku) with Cu Kα radiation (λ = 1.5406 Å). Raman spectroscopy was performed using a Horiba iHR550, utilizing a red laser with a wavelength of 633 nm. Field emission scanning electron microscopy (FE-SEM) was performed using a Supra 35-VP system (Carl Zeiss), operated at 10 kV. The hydrodynamic size, polydispersity index (PdI), and zeta potential of the sample were measured in L.C. Oligo culture medium (AFNOR, 1980) and ultrapure water by dynamic light scattering (DLS) using a Zetasizer Nano ZS90 (Malvern).

To uncover the reactive oxygen species (ROS) emitted by $NiWO_4$ NPs upon exposure to light, a standardized procedure was applied. In this approach, 50.0 mg of the samples were placed in a 50.0 mL Rhodamine B (RhB) solution (1×10^{-5} mol L^{-1}). After subjecting the solution to sonication for proper particle dispersion, it was left in the dark with continuous agitation at 25°C for 30 minutes to establish a molecular adsorptive equilibrium. Following this, UV light lamps (6×15 W, Philips TL-D) were activated, and a sample was withdrawn after 2 h of irradiation. The sample

was then analyzed using a UV-Vis spectrophotometer (V-660 spectrophotometer, JASCO) to track the reduction of the characteristic RhB peak at 554 nm.

This process was iterated four additional times, introducing tert-butyl alcohol (TBA, 99%, Aldrich) to identify ·OH (hydroxyl radicals), silver nitrate ($AgNO_3$, 99.98%, Cennabras) to identify e^- (electrons), ammonium oxalate (AO, 99.9%, Aldrich) to identify h^+ (holes), and *p*-benzoquinone (BQ, 99.9%, Alfa-Aesar) to identify $\cdot O^-$ (superoxide radicals). The decline in photocatalytic efficiency was ascribed to the presence of reactive species, evident in the inhibition of the photocatalytic process.

Field Emission Scanning Electron Microscopy

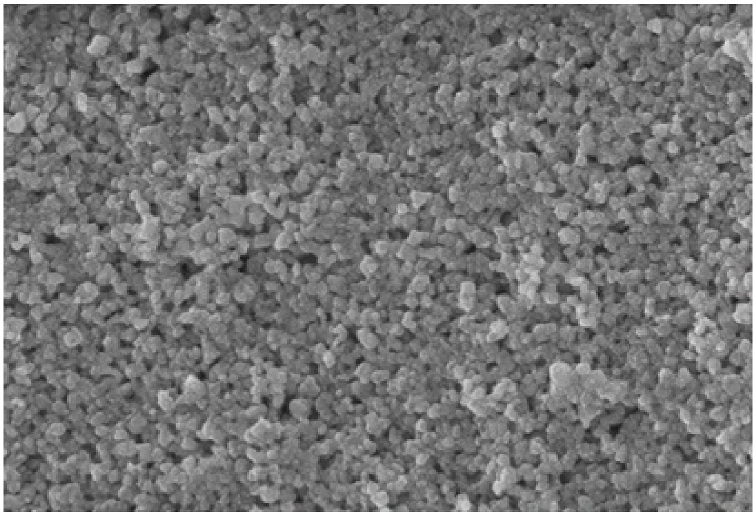

Figure S1: FE-SEM image of $NiWO_4$ nanoparticles.

Surface Gibbs Free Energy

To assess the thermodynamic stability of various terminations of the $NiWO_4$ (110), (111), and (011) surfaces, we employed the *ab initio* atomistic thermodynamics method developed by Rogal and Reuter.[1–5] This section details how we specifically applied this approach to $NiWO_4$. Similar methodologies have been successfully implemented for other ternary oxides in previous studies.[6–9]

The surface free energy per unit area, $\gamma(T, P, \mu_{Ni}, \mu_W, \mu_O)$, is calculated using:

$$\gamma(T, P, \mu_{Ni}, \mu_W, \mu_O) = \frac{1}{2A}[G_{slab}(T, P) - N_{Ni}\mu_{Ni}(T, P) - N_W\mu_W(T, P) - N_O\mu_O(T, P)] \quad \text{(Eq.S1)}$$

In this equation, G_{slab} represents the Gibbs free energy of the symmetric slab; N_{Ni}, N_W, and N_O are the total numbers of Ni, W, and O atoms in the slab, respectively; μ_{Ni}, μ_W, and μ_O denote the chemical potentials of these elements; and A is the surface area.

For our calculations, we approximated the Gibbs free energies (G) with the DFT total energies (E), neglecting zero-point energy and entropy contributions. This approximation is justified because these contributions are relatively small compared to the significant energy differences in solid-state materials, especially when examining different surface morphologies of a single material.[4,6]

Assuming the surface is in equilibrium with bulk $NiWO_4$, the chemical potential of $NiWO_4$ is given by:

$$\mu_{NiWO_4} = \mu_{Ni} + \mu_W + 4\mu_O \quad \text{(Eq. S2)}$$

Since $\mu_{NiWO_4} = E_{bulk}$, substituting Eq. 2 into Eq. 1 yields:

$$\gamma = \frac{1}{2A}[E_{slab} - N_W E_{bulk} - \mu_O (N_O - 4N_W) - \mu_{Ni} (N_{Ni} - N_W)] \quad \text{(Eq. S3)}$$

To prevent the condensation of elemental nickel, tungsten, or oxygen on the surface, the chemical potentials must satisfy:

$$\Delta\mu_O = \mu_O - \frac{1}{2}E_{O_2}^{mol} < 0, \quad \text{(Eq. S4)}$$

$$\Delta\mu_{Ni} = \mu_{Ni} - E_{Ni}^{bulk} < 0, \quad \text{(Eq. S5)}$$

$$\Delta\mu_{W} = \mu_{W} - E_{W}^{bulk} < 0, \qquad \text{(Eq. S6)}$$

where $E_{O_2}^{mol}$, E_{Ni}^{bulk}, and E_{W}^{bulk} are the energies of an O_2 molecule in the gas phase, bulk Ni, and bulk W, respectively. The $\Delta\mu_{i}$ terms represent deviations from the elemental reference states.

By incorporating Eqs. 4 and 5 into Eq. 3, we obtain:

$$\gamma = \theta - \frac{1}{2A}\left[\Delta\mu_{O}\left(N_{O} - 4N_{W}\right) + \Delta\mu_{Ni}\left(N_{Ni} - N_{W}\right)\right] \qquad \text{(Eq. S7)}$$

where

$$\theta = \frac{1}{2A}\left[E_{slab} - N_{W}E_{bulk} - \frac{1}{2}E_{O_2}^{mol}\left(N_{O} - 4N_{W}\right) - E_{Ni}^{bulk}\left(N_{Ni} - N_{W}\right)\right] \qquad \text{(Eq. S8)}$$

The variation in the oxygen chemical potential as a function of temperature and pressure is expressed, assuming ideal gas behavior, as:

$$\Delta\mu_{O}(T,P) = \mu_{O}(T,P) - \frac{1}{2}E_{O_2} = \frac{1}{2}\left[\Delta G_{O_2}^{gas}(T,P^{0}) + kT\ln\left(\frac{P}{P^{0}}\right) + \delta\mu_{O}^{0}\right] \qquad \text{(Eq. S9)}$$

A detailed derivation of this expression can be found in.[8] The values of ΔG_{O_2} are obtained from standard thermodynamic tables,[10] as presented in Table S1. The term $\delta\mu_{O}^{0}$ is a correction factor aligning DFT-calculated energies with experimental data; we use $\delta\mu_{O}^{0} = 0.44$ eV.[7]

The upper limits of the oxygen and nickel chemical potentials are given by Eqs. 4 and 5. The lower bounds are derived by combining Eqs. 4, 5, and 6 with Eq. 2. Since the system is in equilibrium with bulk $NiWO_4$, we have:

$$E_{NiWO_4}^{bulk} = \mu_{Ni} + \mu_{W} + 4\mu_{O} \qquad \text{(Eq. S10)}$$

Introducing the deviations of the chemical potentials, we obtain:

$$\Delta\mu_{W} = E_{NiWO_4}^{bulk} - E_{bulk}^{Ni} - E_{bulk}^{W} - 2E_{mol}^{O_2} - \Delta\mu_{Ni} - 4\Delta\mu_{O} > 0 \qquad \text{(Eq. S11)}$$

Considering that $\Delta\mu_W < 0$ (from Eq. 6), we derive the lower boundary for $\Delta\mu_{Ni}$ and $\Delta\mu_O$:

$$\Delta\mu_{Ni} + 4\Delta\mu_O > E^f_{NiWO_4} \quad \text{(Eq. S12)}$$

where $\Delta E^f_{NiWO_4}$ is the formation energy of $NiWO_4$ from Ni, W, and O_2 in their reference states. In our calculations, we obtained $\Delta E^f_{NiWO_4} = -1.66$ eV/atom, which is consistent with previous computational results.[11]

Work functions for all terminations

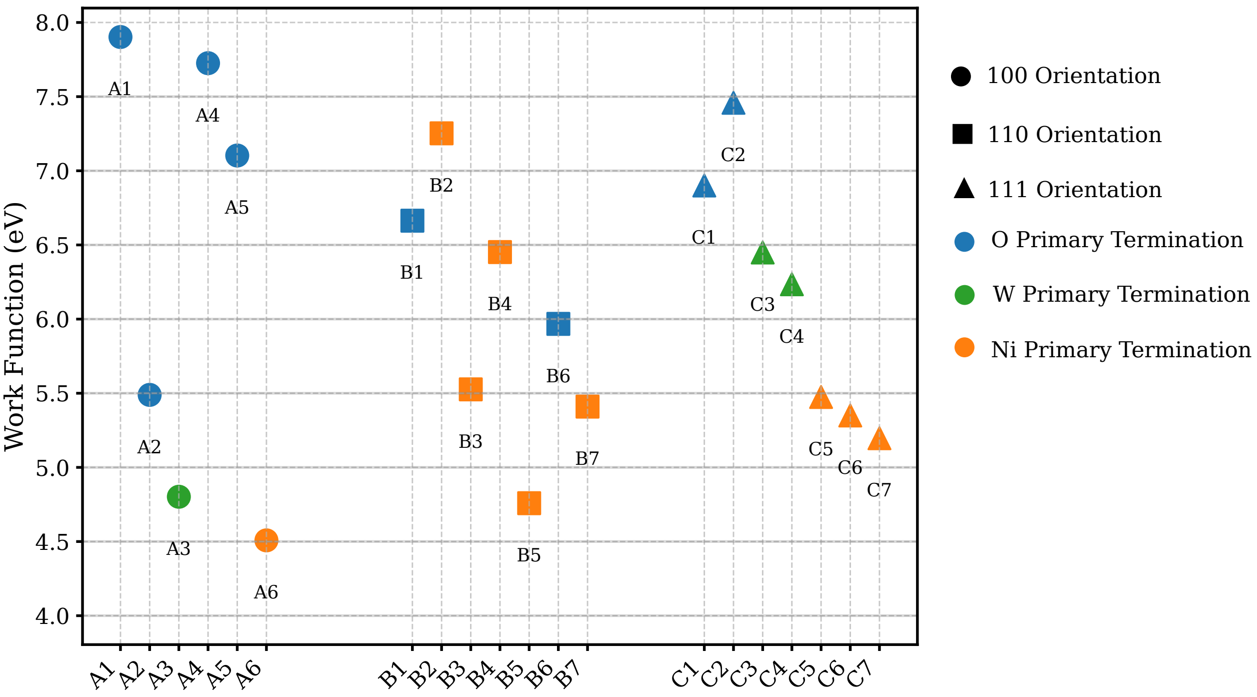

Figure S2: Work functions for all terminations of the (100), (110), and (111) surfaces of $NiWO_4$.

References

(1) Rogal, J.; Reuter, K. Ab Initio Atomistic Thermodynamics for Surfaces: A Primer. *Experiment, modeling and simulation of gas-surface interactions for reactive flows in hypersonic flights* **2007**, *14*, 2–1 – 2–18.

(2) Reuter, K.; Scheffler, M. Composition and Structure of the RuO_2(110) Surface in an O_2 and CO Environment: Implications for the Catalytic Formation of CO_2. *Physical Review B* **2003**, *68*, 045407.

(3) Reuter, K.; Scheffler, M. First-Principles Atomistic Thermodynamics for Oxidation Catalysis: Surface Phase Diagrams and Catalytically Interesting Regions. *Physical Review Letters* **2003**, *90*, 046103.

(4) Reuter, K.; Scheffler, M. Composition, Structure, and Stability of RuO_2(110) as a Function of Oxygen Pressure. *Physical Review B* **2001**, *65*, 035406.

(5) Reuter, K. Ab Initio Thermodynamics and First-Principles Microkinetics for Surface Catalysis. *Catalysis Letters* **2016**, *146*, 541–563.

(6) Poberžnik, M.; Herrero-Saboya, G.; Makovec, D.; Lisjak, D.; Martin-Samos, L. Surface Phase Diagrams of Pristine and Hydroxylated Barium Hexaferrite Surfaces from First-Principles Atomistic Thermodynamics. *Applied Surface Science* **2023**, *637*, 157890.

(7) Cai, Q.; Wang, J.-g.; Wang, Y.; Mei, D. First-Principles Thermodynamics Study of Spinel $MgAl_2O_4$ Surface Stability. *The Journal of Physical Chemistry C* **2016**, *120*, 19087–19096.

(8) Heifets, E.; Ho, J.; Merinov, B. Density Functional Simulation of the $BaZrO_3$ (011) Surface Structure. *Physical Review B* **2007**, *75*, 155431.

(9) Yang, A.; Luo, J.; Xie, Z.; Chen, Q.; Xie, Q. First Principles Calculation of the ZnV_2O_6 (001) Surface Terminations: The Thermodynamic Stability and Electronic Structure Study. *Physical Chemistry Chemical Physics* **2023**, *25*, 12352–12362.

(10) NIST Chemistry WebBook. National Institute of Standards and Technology: Gaithersburg, MD. 2003.

(11) Jain, A.; Ong, S. P.; Hautier, G.; Chen, W.; Richards, W. D.; Dacek, S.; Cholia, S.; Gunter, D.;

Skinner, D.; Ceder, G.; Persson, K. A. Commentary: The Materials Project: A Materials Genome Approach to Accelerating Materials Innovation. *APL Materials* **2013**, *1*, 011002.
